# In the foothill zone—*Sabanejewia balcanica* (Karaman 1922), in the lowland zone—*Sabanejewia bulgarica* (Drensky, 1928): Myth or reality?

**DOI:** 10.1002/ece3.6529

**Published:** 2020-07-03

**Authors:** Peter Križek, Jan Mendel, Jakub Fedorčák, Ján Koščo

**Affiliations:** ^1^ Faculty of Humanities and Natural Sciences Department of Ecology University of Prešov in Prešov Prešov Slovakia; ^2^ Institute of Vertebrate Biology Czech Academy of Sciences Brno Czech Republic

**Keywords:** golden loaches, microhabitat preferences, mitochondrial DNA, morphology, phenotypic plasticity

## Abstract

The status of golden loaches (genus *Sabanejewia*) in the region of Central Europe and Balkans is still ambiguous. The greatest controversy is caused by species *Sabanejewia balcanica* and *S. bulgarica*. Both species are characterized by a wide spectrum of morphological variability and overlapping of distinguishing features, which then lead to difficulties in their determination. Previous phylogenetic studies aimed on the resolving of their taxonomic status did not include samples from their type localities and so led to a lack of their true distribution in this region. Therefore, the main aim of this study was to identify taxonomic status of golden loaches populations in the region of the middle Danube basin and adjacent areas on the model territory of Slovakia. For this purpose, we used novelty approach (morphological, molecular, and microhabitat) and we also included the missing samples from the type localities of both species. Based on mtDNA all the Slovakian samples reflected haplotype richness revealed on the type locality of *S. bulgarica*, although the genetic distances from other representatives of the genus *Sabanejewia* occurring are not significant. Within the morphology, we have revealed a great measure of variability in studied populations, which is largely caused by different habitat conditions and thus representing a phenotypic plasticity of these fish.

## Graphical abstract

The work deals with the ecologic and molecular status of Vladykov's Golden loach in selected European river basins. Our study is the first to report the remarkable results achieved using a new molecular, morphological, and ecological approach, which provides the basis for further studies.

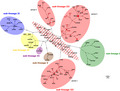

## 1 PREFACE

In the beginning, there was *Cobitis*. In 1929, Vladykov (Coad et al. 1988; McAllister 1988) came here and said: “It's *Sabanejewia*!” Other classic morphologists described several others species and subspecies (Drensky, 1928; Economidis & Nalbant, 1996; Jászfalusi, 1951; Karaman, 1963; Nalbant, 1957; Vasiľeva & Vasiľev, 1988; Witkowski, 1994). Geneticists came and canceled subspecies, some of them promoted under their name to species (Perdices, Doadrio, Economidis, Bohlen, & Bănărescu, 2003). But they did not clarify everything. Recent information on the occurrence of two species—in the foothill zone—*S. balcanica* in the lowland zone—*S. bulgarica*—came from several regions of the Danube basin (Csipkés & Stündl, 2015; Iftime, 2002). Is it myth or reality?

## 2 INTRODUCTION

Systematics of loaches of the genus *Sabanejewia* actually include 10 fish species (Kottelat, 2012), of which eight occur in Europe (Kottelat & Freyhof, 2007; Marešová et al., 2011) and two remaining are widespread in southwestern Asia (Sayyadzadeh, Abbasi, & Esmaeili, 2018). However, in the beginning representatives of this genus were assigned to the related genus *Cobitis*. Until Vladykov (1929) performed a detailed morphological analysis and said: “It's *Sabanejewia!*” But, from of its establishment the genus by itself was questioned. As generally accepted among scientists, the validity of common name *Sabanejewia* has met with recognition until paper published by Nalbant (1963), who acknowledged the Vladykov's claims of significant morphological difference of this genus as justified. For a long time, taxonomy of individual representatives of *Sabanejewia* genus was also complicated. Almost all populations of golden loaches in Europe were perceived as polytypic species *Sabanejewia aurata* (Filippi 1863) (Bănărescu, Nalbant, & Chelmu, 1972). Subsequently, several of its subspecies were described by other classic morphologists (Drensky, 1928; Economidis & Nalbant, 1996; Jászfalusi, 1951; Karaman, 1963; Nalbant, 1957; Vasiľeva & Vasiľev, 1988; Witkowski, 1994).

At the turn of the millennium, application of karyological (Boroń, 2000; Lodi & Marchionni, 1980; Ráb, Roth, & Vasiľeva, 1991; Vasiľeva & Vasiľev, 1988) and biochemical (Ivanova & Dobrovolov, 1999), but mostly molecular research methods (Bartoňová et al., 2008; Buj et al. 2008; Ludwig, Becker, & Bohlen, 2000; Perdices et al., 2003) have brought a progressive shift in systematics and phylogeny of the genus *Sabanejewia*. So far most throughout phylogenetic study based on the mtDNA data (Perdices et al., 2003) identified six main monophyletic lineages inside the genus: *Sabanewia larvata*, *S. romanica*, *S. aurata/S. caucasica*, *S. kubanica*, *S. baltica,* and the Danubian‐Balkanian (DB) complex consisting of six sublineages with a dominant position of species *Sabanejewia balcanica* within them. However, all these studies contain one common deficit. They did not include samples from type localities for the examined species of this study.

**Figure 1:**
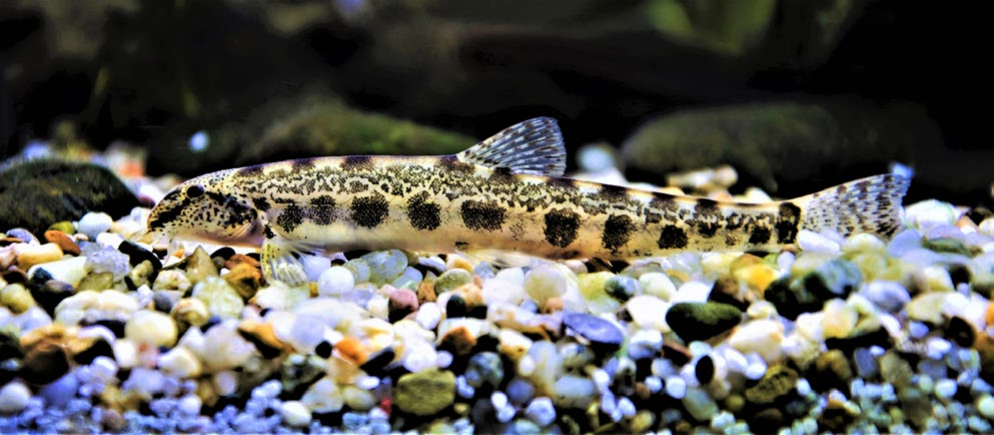
Typical coloration pattern of foothill ecomorph of *Sabanejewia bulgarica*. Specimen from the Kysuca River, Slovakia, male, 72 mm SL; author: Peter Križek

In the region of Central Europe and Balkans, the taxonomic status of *Sabanejewia* populations in Danube basin is still uncertain (Ahnelt & Mikschi, 2004; Erös, Sallai, & Kotusz, 2003; Kováč, 2015; Sály, 2019). The biggest question marks hang over the species *Sabanejewia balcanica* (Karaman 1922) and *S. bulgarica* (Drensky, 1928). The occurrence of both is often reported in the same rivers (Csipkés & Stündl, 2015; Guti & Pekárik, 2016; Iftime, 2002), where the first species prevails in foothill zone, while the latter in their lower parts with sympatric occurrence of both in their contact zone (Bănărescu et al., 1972; Csipkés & Stündl, 2015; Iftime, 2002; Kottelat & Freyhof, 2007; Telcean & Cupșa, 2009). Morphological determination of these species is based only on their coloration pattern (Figure 1) and the difference in relative body depth (Kottelat & Freyhof, 2007). Complications in species recognition and identification are also caused by a presence of morphological intergrades among them in case of their sympatric occurrence (Bănărescu, 1966; Bănărescu et al., 1972; Iftime, 2002).

For these reasons, we decided to examine these irregularities on the model territory of Slovakia, where the status of *Sabanejewia* fish has also been ambiguous. The occurrence of both morphological forms together with their intergradation forms in some localities was recorded in this area. Novomeská and Kováč (2016) state that there is more than one species of *Sabanejewia* occurring in this country. However, these claims are not supported by any further information. Based on the variability of cytochrome *b* gene in samples taken from six Slovakian rivers, Bartoňová et al. (2008) has included them into the sublineages III and IV of the DB complex (Perdices et al., 2003) and concluded that only species *S. balcanica* (Karaman 1922) occur in this territory. However, individuals resembling species *Sabanejewia bulgarica* (Drensky, 1928) by their pigmentation and physical proportions have been recorded in the catchment area of lowland streams in Eastern Slovakia (unpublished data). Some of literature sources (Csipkés & Stündl, 2015; Movchan, 2011; Szepesi & Harka, 2013) report the occurrence of this species near this territory. Kottelat and Freyhof (2007) even mention its occurrence in the Tisza basin and in the Danube itself up to Bratislava (capital city) in Slovakia. This investigation aimed at helping to clarify taxonomic issues, but surely it did not enable complete clarification. Consequently, the main objective of this study was to identify status of fish of the genus *Sabanejewia* in region of the middle Danube basin and adjacent areas on the model of Slovakia simultaneously based on morphological, microhabitat, and molecular approach, which has not been carried out up to present.

## 3 MATERIALS AND METHODS

### 3.1 Study area and samples collecting

Fish from nine sites in the Slovak territory and one site near the town of Vidin, Bulgaria (type locality of *Sabanejewia bulgarica*), were sampled for this study (Table 1). In addition, 14 voucher specimens (catalogue numbers NPM P6V 85,299, 85,303–85,310, and 85,313–85,317) from the river Treska in the City of Skopje, Republic of North Macedonia (close to the type locality of *S. balcanica*) (42°00′07.8″N, 21°20′48.4″E) borrowed from the National Museum in Prague, Czech Republic, were also included for morphological analyses. The selection of the sampling sites in Slovakia covered all main areas of the *Sabanejewia* fish distribution in this country (Koščo et al., 2008). Identification of specimens from Slovakia was based on external morphological characters and coloration patterns as reported by Kottelat and Freyhof (2007).

**Table 1:** Basic information about sampling sites

River	Ni	Nmh	Altitude (m a.s.l.)	Substrate type	Coordinates	Nmt	Cyt*b* haplotypes	Source
Blh	12	12	157	Sand, gravel, pebbles, cobbles, silt	48°56′8.19″N 21°14′53.96″E	6	H55, H56 (3), H57−58	Our data
Bodrog	8	6	97	Clay, sand	48°26′12.88″N 21°49′5.11″E	6	H14−17, H12 (2)	Our data
Danube	8	8	32	Sand, silt	44°0′31.88″N 22°56′30.22″E	10	H7 (2), H12, H20, H41−46	Our data
Ipeľ	13	13	129	Sand, mud	48°4′22.63″N 19°5′16.48″E	12	5,605, 5,607, 5,609–10, 5,612, 5,615–16, 5,623–24, 5,659–60, 5,662	Bartoňová et al. (2008)
Kysuca	21	9	338	Gravel, pebbles, cobbles, boulders	49°16′6.50″N 18°45′7.32″E	6	H1−6	Our data
Laborec	18	18	292	Boulders, gravel, cobbles	49°13′55.99″N 21°53′33.54″E	4	H27 (2), H28−29	Our data
Latorica	26	12	102	Clay, mud	48°28′27.17″N 22°7′10.25″E	8	H7−8, H9 (2), H10−12	Our data
Torysa	17	17	224	Sand, gravel, pebbles, cobbles, silt	48°56′8.19″N 21°14′53.96″E	7	H22 (2), H23, H24 (2), H25−26	Our data
Ulička	17	17	236	Gravel, pebbles, cobbles, boulders	48°56′53.66″N 22°26′17.27″E	4	H18 (2), H19, H21	Our data
Vlára	14	–	235	Boulders, gravel, cobbles	48°58′26.95″N 18°6′20.26″E	9	5,667, 5,669, 5,672, 5,674‐75, 5,678‐79, 6,600‐03	Bartoňová et al. (2008)

Note

Ni = number of individuals evaluated in morphological analyses; Nmh = number of individuals analyzed within microhabitat preferences; Nmt = number of samples included to molecular analyses (number in brackets represents occurrence of haplotypes in case of more than one individual).

During the sampling, selected microhabitat parameters were recorded using point sample method (Copp & Peňáz, 1988) modified according to Pekárik, Koščo, and Švátora (2012). At each sample point, where *Sabanejewia* specimen was present, four microhabitat variables were recorded: water depth to the nearest centimeter; wetted width; average velocity taken in 5‐s interval measured 5 cm above the bottom using of flow probe (Valeport Flow Meter, Valeport Ltd.) and substratum type classified to categories as follows: silt, mud, clay, sand, gravel, pebbles, cobbles, boulders, and bedrock according to Pekárik et al. (2012). Due to the low abundance of *Sabanejewia* specimens at some sampling sites (Bodrog, Kysuca and Latorica Rivers), fish from previous samplings without evaluating the microhabitat parameters were also included to replenish the material for morphological studies.

Immediately after capture fish were anaesthetized, individually labeled and fin clip was taken and stored in 96% ethanol for later molecular analyses. The specimens were placed in labeled plastic bottles and preserved in 6% of formaldehyde solution. Voucher specimens are stored at the Department of Ecology of the University of Prešov (Slovakia).

### 3.2 Morphological analyses

**Figure 2:**
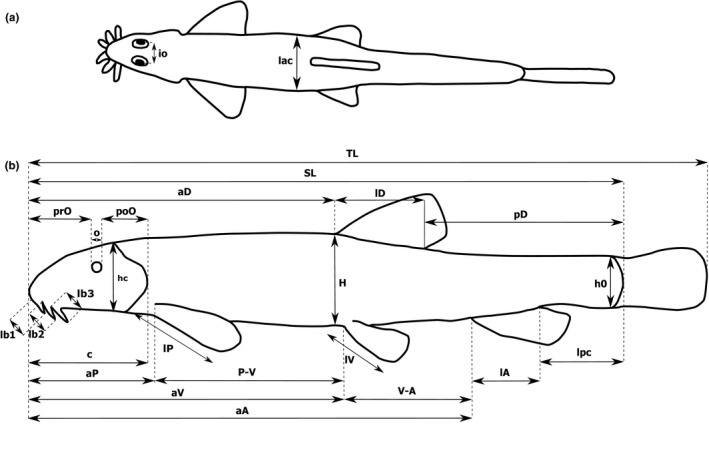
Diagram demonstrating morphometric characters measured. (a) dorsal view and (b) lateral view. Abbreviations: standard length (SL), total length (TL), head length (c), preanal distance (aA), prepelvic (preventral) distance (aV), prepectoral distance (aP), predorsal distance (aD), postdorsal distance (pD), distance between pectoral and ventral fins (P‐V), caudal peduncle length (lpc), length of dorsal (lD), anal (lA), pectoral (lP) and pelvic (lV) fins, maximum body depth (H), minimum body depth (h0), head depth (hc), maximum body width (lac), preorbital distance (prO), postorbital distance (poO), eye diameter (o), distance between eyes (io), length of first (lb1), second (lb2) and third (lb3) pair of barbels

Since a preservation can cause deformations on the body shape and hence to affect final morphological analysis (Sotola et al., 2019), all measurements were taken at least after 3 months of their preservation. To minimize any ontogenetic differences and conservation bias, only well preserved sexually identified adult specimens (SL > 55 mm) (Zanella et al., 2008; own findings) were used for our study. Before each measurement, fish were placed into the cold water for at least 24 hr. Then, a total of 26 morphometric characters (including SL and TL) were measured on the left side of body (Figure 2) to the nearest 0.01 mm using a digital caliper. To avoid any bias, all measurements were made point to point by one author. In order to minimize the resulting measurement error, each measurement was repeated three times and subsequently averaged (Morinaga & Bergmann, 2017). Morphometric characters taken on the body were expressed in percentage (%) of standard length (SL), while measurements on the head in % of head length (c). Caudal peduncle depth (h0) was expressed in % of body depth measured on the basis of dorsal fin (H).

Despite of fact that several significant differences occur between males and females of the genus *Sabanejewia* (Bohlen, 2008; Nalbant, 1963; Vasiľeva & Vasiľev, 1988), there was considerable overlap between both sex groups character ranges.

Moreover, we have assumed a significant impact of local habitat conditions on body shape independent of sex. Therefore, sexual dimorphism was not expected to affect the results.

In addition to morphometric measurements, 12 meristic parameters were counted (Table 6). Fin rays were counted under the light microscope with sufficient zoom. The last two unbranched rays in dorsal and anal fin, which articulate on single pterygiophore, were counted as “1^1^/_2._”

### 3.3 Molecular analysis

Total genomic DNA was extracted from a small piece of the pectoral fin by a commercial kit (GT300, Geneaid). The entire sequences of cytochrome *b* (1,140 bp) were amplified by polymerase chain reaction (PCR) with primer pair GluDG.L (Palumbi, 1996) and H16460 (Perdices & Doadrio, 2001). PCRs were performed in 25 µl volume in Mastercycler Pro (Eppendorf) by the help of a commercial kit (PPP Master Mix; Top‐Bio) according to the manufacturer's instructions. The thermal cycling consisted of one initial cycle of denaturation at 94°C for 1 min, followed by 30 cycles of denaturation at 94°C for 15 s, annealing at 60°C for 15 s, extension at 72°C for 1 min, and a final extension at 72°C for 7 min. The amplicons were visualized by gel electrophoresis using Midori Green Advance and 1.7% agarose gels. The PCR products were purified using DNA Clean & Concentrator‐5 Kit (Zymo Research). Sequencing was performed using a commercial service (Macrogen, Europe). All PCR amplicons were sequenced from both directions to ensure high quality reads. The DNA sequences were edited and aligned using the Seqman module within Lasergene 15.0 (DNASTAR Inc.) and also checked manually. The mentioned above genetic analyses were carried out as a DNA service by IVB AS (Institute of Vertebrate Biology of the Czech Academy of Sciences, Brno, Czech Republic). The sequences were deposited in the GenBank database under Accession Nos. MN149863‐901. In addition, the sequences of other specimens included to the Danubian‐Balkanian complex (Bartoňová et al., 2008; Marešová et al., 2011; Perdices et al., 2003) were added for comparison with our samples. Detail list of all studied taxa, their haplotype classification to sampling sites, haplotype frequencies, and GenBank accession numbers are shown in Table S1.

### 3.4 Data analysis

Multivariate normality was tested by visualization of morphometric variables (MVs) through the histograms and Mahalanobis multivariate QQ‐plot. Before analyzing, morphometric dataset was standardized by arcsine square root transformation in terms of its percentage character.

1. Principal component analysis (PCA) using the method of correlation matrix was conducted to reveal an overall pattern of morphological variation. The number of PCA axes important for interpretation was tested by function of broken‐stick model. Significance of interpopulation differences was evaluated by pairwise permutational multivariate analysis of variance (PERMANOVA) with 999 permutations. Family‐wise errors were corrected by false discovery rate correction (FDR) for each pair.
2. Cross‐validated discriminant function analysis (DFA) was used to test whether the examined individuals were correctly classified into the certain population.
3. To test whether the set of environmental variables (EVs) significantly influences the overall body shape of analyzed fish, linear redundancy analysis (RDA) was used. Matrix of MVs was overlaid by dataset of EVs, and function of environmental vector fitting (envfit) with 999 permutations was used to test, which EVs have significant effect for distinguishing morphotypes of evaluated fish groups. The same approach was assessed with a set of coloration (number of lateral and dorsal spots) data.

All statistical analyses were performed in R statistical software ver. 3.5.2 (R Core Team, 2019) using functions of packages morphoTools (Koutecký, 2014), MASS (Venables & Ripley, 2002), vegan (Oksanen et al., 2013), and pairwiseAdonis (Arbizu, 2017). Visualization of PCA scatterplot was conducted by functions of package ggplot2 (Wickham, 2016).

For phylogenetic reconstructions and delimitation of boundaries within the DB complex, all forward and reverse sequences were assembled, edited, and aligned using the Seqman module (Lasergene v15) and also were checked by eye. Furthermore, as a final quality control, cyt b sequences were translated to verify that they were free of stop codons, frame‐shifts, and gaps. The genetic dataset was analyzed by Bayesian inference (BI) using MrBayes 3.1.2 (Ronquist et al. 2012), the maximum‐likelihood (ML) method using PhyML (Guindon et al., 2010), and neighbor‐joining algorithm (NJ) using PAUP* 4.0B.10 (Swofford, 2002). The best‐fit model of molecular evolution was determined for mitochondrial dataset using the Akaike Criterion (AIC) in Modeltest ver. 2.1.4 (Posada, 2008). MrBayes was run with six substitution types (nst = 6) and considered gamma‐distributed rate variation and the proportion of invariable positions (GTR + G + I). For BI, we ran four simultaneous Monte Carlo Markov Chain (MCMC) for two million generations and sample frequency every 100 generations. The first 5,000 trees were excluded as burn‐in. The remaining trees were used to compute a 50% majority rule consensus tree. For ML analysis, we conducted heuristic searches under a GTR + I + G. For NJ analysis, DNA distance was calculated using MEGA 7 (Kumar, Stecher, & Tamura, 2016). Robustness of inferred trees was assessed by bootstrapping (1,000 replicates) in ML or NJ analyses and posterior probability values in BI analysis. Branch support values were evaluated in accordance with Yang, He, Freyhof, Witte, and Liu (2006), where good support was defined as bootstrap values of 75%–88% and posterior probabilities of 85%–94%, strong support as bootstrap values of 89%–100% and posterior probabilities of 95%–100%. Haplotype network was constructed to estimate the genealogical intraspecific relationships employing the statistical parsimony (Clement, Snell, Walke, Posada, & Crandall, 2002; Templeton, Crandall, & Sing, 1992) implemented into the PopArt software (Leigh & Bryant, 2015). A 95% connection limit was calculated. A number of polymorphic sites (S), nucleotide diversity (π), haplotype diversity (Hd), and neutrality tests were calculated using DnaSP 6 (Rozas et al., 2017). The global cyt b dataset was also analyzed using three analytical methods—Poisson Tree Processes (bPTP, Zhang, Kapli, Pavlidis, & Stamatakis, 2013), multi‐rate PTP (mPTP, Kapli et al., 2017), and Bayesian clustering (STRUCTURE, Pritchard, Stephens, & Donnelly, 2000) to correct delimitation of groups and sublineages of the DB complex. The PTP delimits group boundaries based on rooted phylogenetic trees with speciation and branching events modeled by maximum‐likelihood and Bayesian support examining the number of substitutions. This model has been integrated with the evolutionary placement algorithm (EPA‐PTP) to estimate the number of groups in phylogenetic placements. The both PTP analyses were performed first by generating a ML tree in MEGA7 and then exporting the tree as a Newick file, which was subsequently used in an online version of bPTP (http://species.h‐its.org/ptp/; 500,000 generations with a thinning of 500 and a burn‐in of 0.1) and mPTP in standalone version (http://github.com/Pas‐Kapli/mptp; four simultaneous MCMC runs of 10 million generations, sampling every 10,000 steps). The PGDSpider (Lischer & Excoffier, 2012) was used as a conversion tool for population genetics formats (sequences/binary markers). An unbiased Bayesian approach using MCMC clustering of samples was conducted via the STRUCTURE v2.2.3 software. Parameters were set as for SNPs data for each individual and assessed for values of *K* ranging from 1 to 17. Burn‐in and MCMC iteration settings were 50,000 and 100,000, respectively. Allele frequencies were treated as correlated. For each value of *K*, six replicate simulations were conducted with admixture model without using population prior (LOCPRIOR) information. The results were analyzed via Clumpak program (Kopelman, Mayzel, Jakobsson, Rosenberg, & Mayrose, 2015) and the Δ*K* statistics (the second order rate of change in log probability [Ln Pr(*X*/*K*)] between successive values of *K*) was calculated using STRUCTURE Harvester v0.6.94 (Earl, 2012) as per Evanno, Regnaut, & Goudet, 2005.

## 4 RESULTS

The mean values and standard deviations of morphometric characters expressed in relations to SL, H, and c for studied populations are listed in Table 2. Broken‐stick model detected first component axis to be suitable for PCA interpretation, since its percentage of explained variation was higher than broken‐stick percentage. The first principal component (PC1) accounted for 49.5%, while the second (PC2) for 10.5% of the total variance explained. Morphometric characters with the highest absolute correlation were lengths of lb1, lb2, and lb3 to the first and hc, io, and aA to the second axis, respectively (Table 3).

**Table 2:** Mean values of morphometric characters for studied populations

Population	Blh		Bodrog		Danube		Ipeľ		Kysuca		Laborec		Latorica		Torysa		Treska		Ulička		Vlára	
Character	Mean	*SD*	Mean	*SD*	Mean	*SD*	Mean	*SD*	Mean	*SD*	Mean	*SD*	Mean	*SD*	Mean	*SD*	Mean	*SD*	Mean	*SD*	Mean	*SD*
TL	91.32	‐	77.55	‐	78.18	‐	73.13	‐	90.29	‐	80.98	‐	81.32	‐	84.75	‐	78.52	‐	86.78	‐	90.94	‐
SL	78.42	‐	66.76	‐	67.00	‐	62.81	‐	76.91	‐	69.96	‐	69.94	‐	74.21	‐	67.15	‐	74.39	‐	77.78	‐
In % SL																						
c	20.14	0.52	21.68	0.81	21.44	0.48	21.44	1.05	20.02	0.73	19.43	0.76	20.67	0.77	20.78	0.75	20.64	0.81	19.64	0.62	19.99	0.75
aA	73.38	1.64	75.27	1.28	74.13	0.93	73.45	1.07	72.88	1.44	71.97	1.26	74.74	1.42	73.68	1.68	76.29	1.11	72.72	1.07	73.66	1.43
aV	48.45	1.15	50.26	1.70	48.87	0.69	49.29	0.99	48.60	1.17	48.45	1.45	50.05	1.13	49.52	1.22	50.60	1.43	48.82	0.92	49.82	1.46
aP	20.82	0.72	22.35	1.43	22.84	0.96	23.21	1.03	20.53	0.85	21.81	0.75	21.79	1.09	22.80	0.87	21.78	0.63	21.79	1.07	21.22	0.87
aD	47.70	1.52	50.43	1.58	49.48	1.32	50.04	1.47	47.81	1.26	46.47	1.64	49.48	1.34	48.03	1.36	49.29	1.26	46.93	0.94	48.00	1.00
pD	43.77	0.87	39.63	1.47	41.96	0.80	42.41	1.20	43.61	0.78	44.65	1.01	40.64	1.66	42.98	0.98	42.52	0.93	44.06	0.74	42.67	1.06
P‐V	29.20	1.53	29.58	2.06	29.29	0.95	28.79	0.66	30.09	1.34	30.67	1.24	30.94	1.08	30.39	1.19	30.13	1.17	29.40	0.92	30.65	1.13
V‐A	26.67	1.27	26.03	1.60	26.25	0.94	25.37	0.97	25.57	1.13	24.23	1.10	26.47	1.25	25.44	1.15	26.71	0.72	24.99	1.05	25.75	1.13
Lpc	19.92	1.03	17.92	1.74	18.35	1.05	19.33	0.98	20.22	1.34	21.08	1.38	18.34	1.19	19.00	1.23	18.17	1.05	20.52	1.03	19.54	0.97
lD	10.43	0.67	10.38	0.79	10.17	0.91	9.99	0.74	10.68	0.65	9.81	0.56	10.23	0.53	10.42	0.58	10.17	0.80	9.98	0.63	10.48	0.51
lA	8.67	0.69	8.34	0.66	8.20	0.67	7.93	0.66	8.45	0.79	7.68	0.67	8.42	0.67	8.22	0.69	7.55	0.53	8.01	0.78	8.37	0.65
lP	14.97	0.76	15.38	1.21	15.17	0.80	15.75	0.87	14.43	0.84	14.65	0.70	15.11	0.66	15.29	0.54	15.28	0.75	14.76	0.94	14.67	1.35
lV	13.13	0.65	13.08	0.66	12.88	0.37	13.78	0.97	12.66	0.67	12.78	0.51	13.04	0.60	13.34	0.70	12.77	0.76	13.04	0.65	12.99	1.09
H	15.99	0.67	15.62	0.71	15.85	0.89	15.25	0.90	16.19	0.92	14.69	0.81	15.93	0.77	15.51	0.55	16.23	1.22	15.17	0.84	15.11	0.61
lac	10.57	0.91	10.17	0.80	9.57	1.21	9.35	0.70	11.03	1.35	9.24	0.90	9.90	0.96	9.59	0.88	10.80	1.09	10.15	0.99	9.47	0.64
In % H																						
h0	52.08	2.02	51.05	2.92	46.66	1.84	52.25	2.34	51.45	2.39	51.38	2.56	50.45	2.64	54.62	1.75	48.27	2.95	52.03	3.28	54.64	2.09
hc	64.82	2.99	64.67	4.18	62.19	2.38	62.27	2.70	63.82	2.10	63.54	2.76	64.93	3.73	64.66	3.10	63.03	2.27	65.65	2.19	65.75	2.24
In % c																						
prO	46.23	2.20	44.79	2.04	45.69	1.82	47.82	1.56	47.14	2.14	47.55	1.71	45.15	2.10	48.20	2.13	47.48	2.70	48.51	3.14	46.27	1.23
poO	52.27	1.84	51.74	2.78	52.22	2.43	50.85	2.80	51.84	1.94	49.59	1.71	53.73	1.98	50.82	2.16	47.32	3.36	51.12	2.07	51.91	1.46
io	25.98	2.07	23.55	2.22	24.44	2.09	24.61	1.18	26.10	1.83	25.15	1.87	24.87	2.00	24.15	1.78	22.51	2.20	25.66	2.46	25.43	1.44
o	15.45	0.94	15.04	0.79	15.03	1.65	16.06	0.88	15.04	1.06	15.96	0.77	15.87	1.53	14.60	0.83	17.14	1.64	16.28	1.13	13.95	1.31
lb1	18.82	2.66	19.02	4.94	20.28	2.03	14.63	2.24	16.81	2.28	9.36	2.11	19.54	1.81	17.81	2.45	17.06	2.36	16.47	2.88	12.02	1.75
lb2	23.01	2.39	25.14	2.82	24.47	2.98	22.50	2.30	21.53	1.97	14.34	2.15	25.50	2.65	23.66	2.39	22.27	2.57	21.62	3.38	17.44	2.01
lb3	26.27	2.20	27.91	2.60	26.67	1.93	24.61	3.13	24.06	2.07	18.14	2.41	26.52	3.60	27.01	2.43	23.72	2.89	24.48	3.93	16.61	2.76

**Table 3:** Loadings of the first two principal components derived from PCA

Character	Component	
	PC1	PC2
c	−0.051	0.190
aA	−0.077	0.233
aV	−0.026	0.174
aP	−0.021	0.200
aD	−0.079	0.170
pD	0.087	−0.168
P‐V	0.007	0.024
V‐A	−0.065	−0.019
lpc	0.098	−0.164
lD	−0.032	−0.029
lA	−0.042	−0.112
lP	−0.043	0.043
lV	−0.026	0.028
H	−0.067	−0.041
lac	−0.068	−0.162
h0	0.056	−0.167
hc	−0.038	−0.578
prO	0.042	−0.063
poO	−0.096	−0.284
io	−0.013	−0.482
o	−0.023	−0.065
lb1	−0.596	−0.122
lb2	−0.541	−0.002
lb3	−0.533	0.106

**Figure 3:**
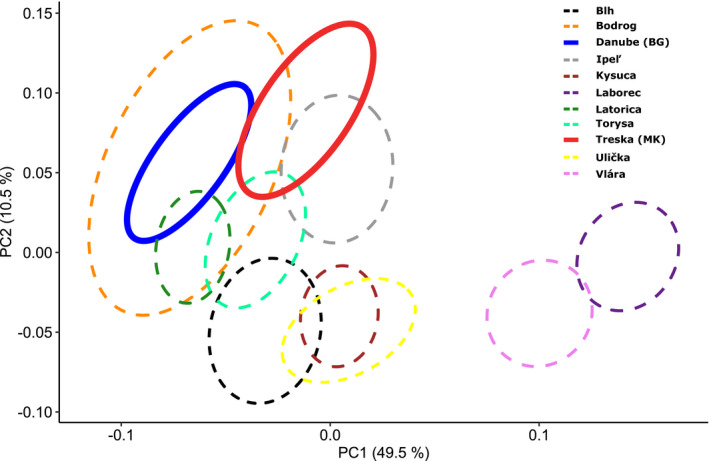
Scatterplot of principal component analysis (PCA). Ellipses represent 95% confidence intervals of the specimens classified into the certain population; Slovak populations are shown as dashed lines; populations from type localities for *S. balcanica* (Treska, MK) and *S. bulgarica* (Danube, BG) are shown as bold solid lines

The scatterplot of PCA showed obvious morphological variation especially in Slovakian samples compared to both *S. balcanica* and *S. bulgarica* populations from *terra typica*. Plotting individual populations based on 95% confidence intervals (Figure 3) showed almost complete overlap of populations from large lowland rivers (Bodrog and Latorica Rivers) together with population of Danube River (Bulgaria) representing *S. bulgarica* morphotype. In the positive direction of PC1 and also in negative direction of PC2, there is a certain trend of clinal transition from large lowland rivers to streams and rivers in submountain zone. Especially, populations from Laborec and Vlára Rivers showed a significant difference from the others. Result of pairwise PERMANOVA confirmed highly significant differences between most of the observed populations (Table 4).

**Table 4:** Pairwise comparisons of permutational multivariate analysis of variance (PERMANOVA) between studied populations based on their morphometric characters

Pairwise comparison	*F*. model	*R* ^2^	Adjusted *p*‐value	Pairwise comparison	*F*. model	*R* ^2^	Adjusted *p*‐value
**Blh vs. Bod**	**3.09**	**.15**	**.005**	**Ipe vs. Lab**	**31.25**	**.52**	**.001**
**Blh vs. Dan**	**4.31**	**.18**	**.001**	**Ipe vs. Lat**	**11.33**	**.23**	**.001**
**Blh vs. Ipe**	**7.14**	**.24**	**.001**	**Ipe vs. Tor**	**5.52**	**.16**	**.001**
Blh vs. Kys	2.75	.08	.015	**Ipe vs. Tre**	**5.49**	**.19**	**.001**
**Blh vs. Lab**	**48.65**	**.63**	**.001**	**Ipe vs. Uli**	**3.41**	**.11**	**.006**
**Blh vs. Lat**	**4.17**	**.10**	**.001**	**Ipe vs. Vla**	**20.17**	**.45**	**.001**
**Blh vs. Tor**	**3.69**	**.12**	**.001**	**Kys vs. Lab**	**45.35**	**.55**	**.001**
**Blh vs. Tre**	**6.98**	**.24**	**.002**	**Kys vs. Lat**	**12.70**	**.22**	**.001**
Blh vs. Uli	2.85	.10	.016	**Kys vs. Tor**	**8.01**	**.18**	**.001**
**Blh vs. Vla**	**30.41**	**.56**	**.001**	**Kys vs. Tre**	**7.90**	**.20**	**.001**
Bod vs. Dan	1.47	.09	.166	Kys vs. Uli	2.18	.06	.049
**Bod vs. Ipe**	**4.93**	**.21**	**.002**	**Kys. vs. Vla**	**24.95**	**.43**	**.001**
**Bod vs. Kys**	**7.24**	**.21**	**.001**	**Lab vs. Lat**	**84.83**	**.67**	**.001**
**Bod vs. Lab**	**38.87**	**.62**	**.001**	**Lab vs. Tor**	**60.20**	**.65**	**.001**
Bod vs. Lat	1.16	.03	.306	**Lab vs. Tre**	**35.51**	**.56**	**.001**
Bod vs. Tor	2.82	.11	.016	**Lab vs. Uli**	**29.44**	**.47**	**.001**
**Bod vs. Tre**	**4.20**	**.19**	**.005**	**Lab vs. Vla**	**9.68**	**.24**	**.001**
**Bod vs. Uli**	**5.58**	**.20**	**.001**	**Lat vs. Tor**	**6.70**	**.14**	**.001**
**Bod vs. Vla**	**24.44**	**.55**	**.001**	**Lat vs. Tre**	**9.27**	**.20**	**.001**
**Dan vs. Ipe**	**8.20**	**.29**	**.001**	**Lat vs. Uli**	**10.79**	**.21**	**.001**
**Dan vs. Kys**	**9.17**	**.25**	**.001**	**Lat vs. Vla**	**48.35**	**.56**	**.001**
**Dan vs. Lab**	**51.77**	**.67**	**.001**	**Tor vs. Tre**	**8.51**	**.24**	**.001**
Dan vs. Lat	2.40	.07	.025	**Tor vs. Uli**	**4.72**	**.13**	**.002**
**Dan vs. Tor**	**6.17**	**.20**	**.001**	**Tor vs. Vla**	**36.75**	**.56**	**.001**
**Dan vs. Tre**	**5.64**	**.23**	**.001**	**Tre vs. Uli**	**5.38**	**.17**	**.001**
**Dan vs. Uli**	**7.09**	**.23**	**.001**	**Tre vs. Vla**	**23.57**	**.50**	**.001**
**Dan vs. Vla**	**35.85**	**.63**	**.001**	**Uli vs. Vla**	**17.77**	**.38**	**.001**
**Ipe vs. Kys**	**7.27**	**.19**	**.001**				

Note

Significant results are marked bold.

Abbreviations: Blh, Blh River; Bod, Bodrog River; Dan, Danube River (BG); Ipe, Ipeľ River; Kys, Kysuca River; Lab, Laborec River; Lat, Latorica River; Tor, Torysa River; Tre, Treska River (MK); Uli, Ulička River; Vla, Vlára River.

Based on the DFA results, the overall assignment of specimens into their original population was 71.5%. The highest proportion of correctly classified individuals into their original group was observed in Laborec and Vlára Rivers (both equally 100%), indicating high difference from the other ones. On the contrary, the lowest number of individuals was correctly included within populations of Bodrog and Blh Rivers (37.5% and 41.7%, respectively) (Table 5). In most cases, the remaining individuals were classified into the populations from sites with similar habitat conditions.

**Table 5:** Percentiles and predicted groups memberships of correctly classified individuals to studied populations assessed by cross‐validated discriminant function analysis (DFA)

Population	% correct	Predicted group membership										
		1	2	3	4	5	6	7	8	9	10	11
Blh (1)	41.7	5	0	0	1	5	0	1	0	0	0	0
Bodrog (2)	37.5	0	3	0	0	0	0	5	0	0	0	0
Danube (3)	87.5	0	0	7	0	0	0	1	0	0	0	0
Ipeľ (4)	76.9	0	0	1	10	0	0	0	2	0	0	0
Kysuca (5)	57.1	3	0	0	0	12	0	0	1	2	2	1
Laborec (6)	100.0	0	0	0	0	0	18	0	0	0	0	0
Latorica (7)	77.0	0	3	0	0	3	0	20	0	0	0	0
Torysa (8)	58.8	1	0	0	3	1	0	0	10	0	2	0
Treska (9)	91.7	0	0	0	0	0	0	0	0	11	1	0
Ulička (10)	58.8	1	0	0	1	0	2	0	3	0	10	0
Vlára (11)	100.0	0	0	0	0	0	0	0	0	0	0	14

### 4.1 Meristic and coloration

The number of fin rays did not show any significant differences between studied populations. Their number was almost constant with only minimal differences (Table 6). Based on coloration, two main groups of fish were formed. Populations from larger lowland streams (Bodrog, Danube, Latorica) were set aside, where the number of lateral and dorsal spots was significantly lower than in others. However, great differences in number of spots were also found in individuals from the same populations (Table 6).

**Table 6:** Meristic characters of analyzed populations

Population	Fin rays					Number of spots		
	P	V	D	A	C	Left	Right	Dorsal
Blh	I/7–9	II/6 (7)	II‐III/ (6.5)7.5	II‐III/5.5	14–15	9–15	11–16	8–13
Bodrog	I/8–9	I‐II/(5) 6	III/6.5–7.5	III/(4.5) 5.5	(13) 14 (15)	6–11	7–9	7–8
Danube	I/8–9	(I) II/6 (7)	III/7.5 (8.5)	III/5.5–6.5	14 (15)	8–13	8–12	7–10
Ipeľ	I/7–9	I‐II/5–6	III/7.5	II‐IV/5.5–6.5	(13) 14–15	11–16	12–16	9–12
Kysuca	I/7–9	II/5–6 (7)	II‐III/6.5–7.5	II‐III/(4.5) 5.5	13–15	8–13	9–13	9–13
Laborec	I/7–9	(I) II/6	II‐III/(6.5) 7.5	II‐III (IV)/4.5–5.5	14–15	9–19	11–19	10–14
Latorica	I/7–9	II/6 (7)	II‐III/6.5–7.5	II‐III/ (4.5) 5.5	(13) 14–15	5–10	7–11	7–10
Torysa	I/7–8	I‐II/5–6	II‐III/6.5–7.5	(II) III (IV)/5.5	(13) 14–15	9–18	10–15	10–13
Treska	I/6–8	II/5–6	III/7.5	III/5.5	(13) 14	9–16	10–16	10–14
Ulička	I/7–8	I‐II/6–7	III/7.5	II‐III/5.5	13–15	10–16	10–16	10–14
Vlára	I/7–9	(I) II/5–6	II‐III/6.5–7.5	III/5.5 (6.5)	(13) 14–15	9–14	10–13	9–13

Note

P = pectoral fin, V = ventral fin, D = dorsal fin, A = anal fin, C = caudal fin; Roman numerals = number of spines, Arabic numerals = number of soft rays (the value in brackets indicates a rare number).

### 4.2 Microhabitat preferences of morphotypes

The RDA model significantly explained (*F* = 43.49, *df* = 1, *p* < .01, 999 permutations) 27.4% of the total variability for the first axis, while the second axis (*F* = 6.26, *df* = 1, *p* = .15, 999 permutations) accounted for only 3.9% of the total model variance. Using envfit function, nine variables were identified to have a significant effect on morphometric dataset (Table 7). In case of the coloration dataset, the result of permutation test has revealed seven significant EVs (Table 7), while also only the first axis (32.4% of total model variance) was important for interpretation (*F* = 51.31, *df* = 1, *p* < .01). The second axis (1.6% of total model variance) was insignificant (*F* = 2.52, *df* = 1, *p* ≥ .98).

**Table 7:** Importance of environmental variables used in RDA analysis

Env. variable	Morphometric dataset				Coloration dataset			
	RDA1	RDA2	*R* ^2^	*p*‐value	RDA1	RDA2	*R* ^2^	*p*‐value
**Depth**	0.850	0.527	.23	<.001***	0.979	0.206	.18	<.001***
Velocity	0.944	0.331	.02	>.38	0.058	0.998	.01	>.69
**Width**	0.685	0.729	.20	<.001***	0.997	0.081	.10	<.01**
Silt	0.828	−0.560	.09	<.01**	0.965	0.263	.00	>.87
Mud	0.122	0.993	.03	>.20	0.973	0.229	.03	>.23
**Clay**	0.859	0.513	.14	<.01**	0.996	0.091	.19	<.001***
**Sand**	0.780	0.626	.22	<.001***	0.738	0.674	.11	<.001***
**Gravel**	−0.773	−0.634	.15	<.001***	−0.940	−0.342	.09	<.01**
Pebbles	−0.049	−0.999	.14	<.001***	−0.854	−0.520	.05	>.10
**Cobbles**	−0.354	−0.935	.08	<.01**	−0.703	−0.711	.10	<.001***
**Boulders**	−0.998	−0.063	.27	<.001***	−0.947	−0.320	.10	<.01**
Bedrock	−0.552	0.834	.01	>.78	0.580	0.814	.03	>.25

Note

Significant variables for both models are marked bold.

*** *p* value < .001

** *p* value < .01

* *p* value < .05

**Figure 4:**
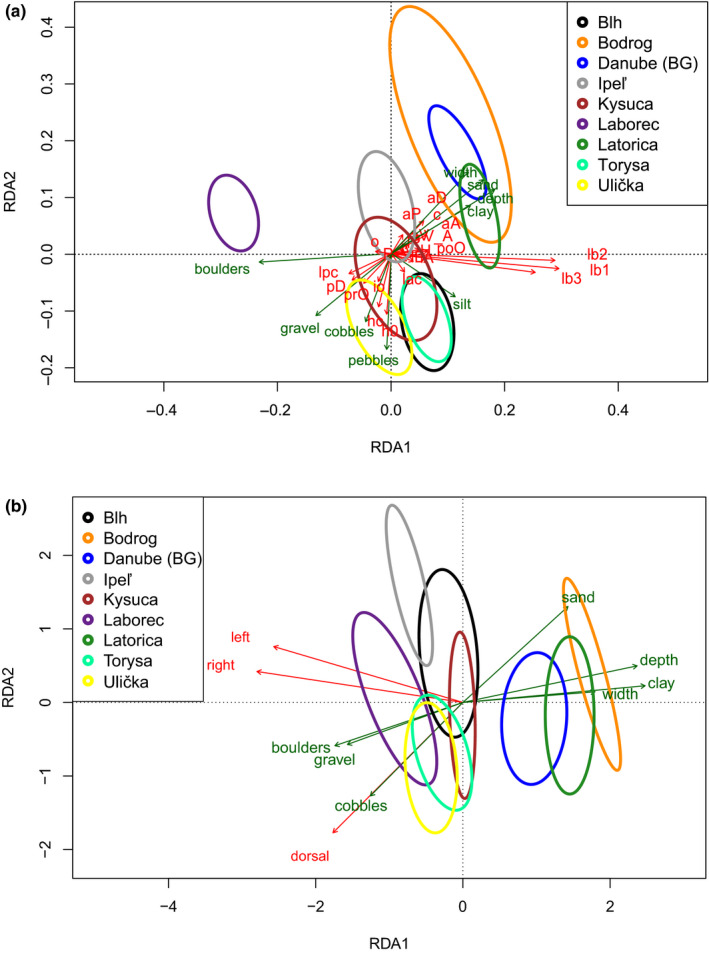
RDA triplot (a) based on morphometric characters and (b) on coloration dataset

Based on final triplots (Figure 4), the occurrence of *bulgarica*‐like morphotype is associated with deeply parts of large rivers and fine substrate (sand, clay, or silt). On the other hand, with thicker substrate (gravel, cobbles, pebbles, and boulders) morphotype of *S. balcanica* prevails. Similar result is observed in coloration pattern, where number of spots decreasing toward larger lowland streams representing a typical habitat for *S. bulgarica*.

### 4.3 Haplotype richness—haplotype network

**Figure 5:**
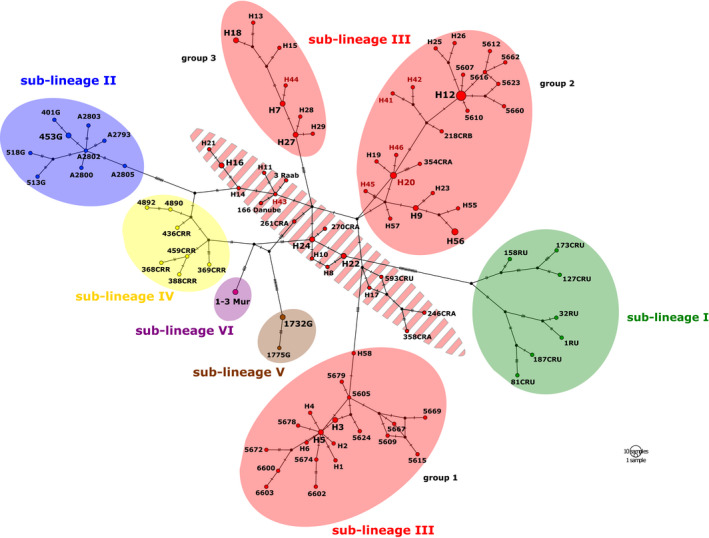
The unrooted TCS haplotype network for the sublineages I‐VI of the Danubian–Balkanian complex based on sequences of the cyt *b*. The haplotype numbers refer to Table S1. The node sizes are proportional to haplotype frequencies. Haplotype numbers from type locality in Vidin, Bulgaria, are highlighted in red

Analysis of mitochondrial sequences from 114 individuals identified 94 cyt b haplotypes based on 135 variable nucleotide and 87 parsimony informative sites. Overall, haplotype diversity was high (0.994 ± 0.003) with relatively low nucleotide diversity (0.0127 ± 0.0008). Genetic diversity indices and the results of neutrality tests of each network section are shown in Table S2. Tajima's D and Fu & Li's D values were negative for all network sections (with *n* > 4) but statistically not significant, indicating an excess of low frequency polymorphisms relative to expectation. The mitochondrial network (Figure 5) has confirmed six main sublineages of DB complex as reported by Perdices et al. (2003) and reflects diversification and haplotype richness within it. The schematic diagram constructed on the basis of statistical parsimony showed a complex pattern of mutual relations within sublineage III of DB complex. The structure of the whole DB complex, including delimitation of individual groups of sublineage III (groups 1–4), was further verified also by phylogenetic and delimitation analyses.

### 4.4 Phylogenetic analyses

**Figure 6:**
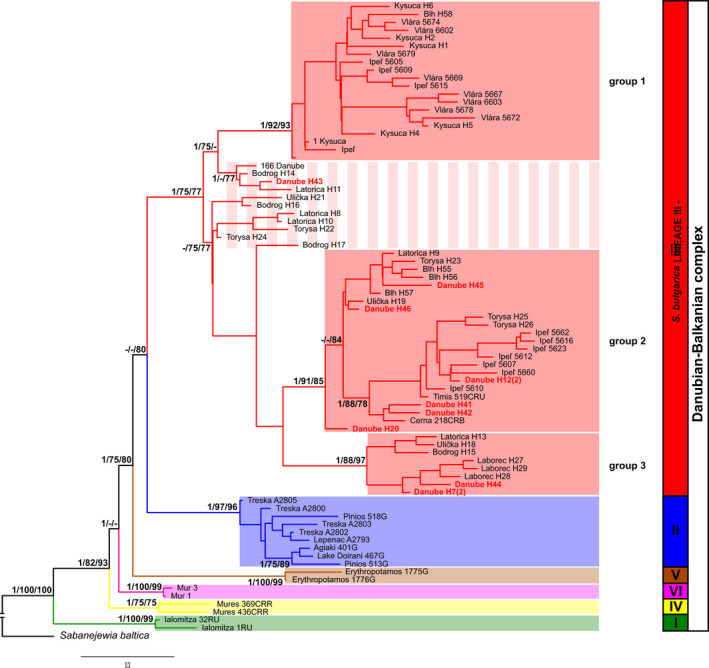
Bayesian consensus tree resulting from the analysis of the cyt *b* data in studied golden loaches taxa with Bayesian posterior probabilities/ML bootstrap/NJ bootstrap values listed near the nodes. Only values > 75% are shown. Haplotype numbers from type locality in Vidin, Bulgaria, are highlighted in red

Phylogram (Figure 6) based on Bayesian inference points to the monophyletic character of DB complex, where the *S. vallachica* represents the most divergent species within the complex. In all the methods revealing the phylogeny of DB complex, the sublineages have a good‐strong bootstrap support values (75%–100%) with a significant Bayesian posterior probabilities. The results confirm the valid species recognized by the scientific community including both investigated species *S. balcanica* and *S. bulgarica* and, besides that, they more precisely define the areas of occurrence in compliance with haplotype profiles of the individuals from both the type localities. Individuals from Slovakia showed a high degree of variability reflecting the haplotype richness revealed on the type locality of *S. bulgarica* in Vidin (Bulgaria) and so forming the mentioning sublineage III of the DB complex.

Based on final phylogram (Figure 6), all the Slovakian samples can be subdivided into two clusters. First one includes samples from western part of Slovakia (Kysuca, Vlára Rivers), while the second consists of individuals from the its eastern part (Bodrog, Torysa, Laborec, Latorica, Ulička Rivers). In both of them, we can find a representative from the middle part of territory (Ipeľ, Blh Rivers). In a more detailed sense, dataset of Slovakian samples can also be subdivided into three groups with a strong statistically support: Group 1 mainly formed by samples from Kysuca and Vlára Rivers (western part of Slovakia), group 2 mostly formed by individuals from Blh, Ipeľ, and Torysa Rivers (predominantly middle part of the country), and group 3 consisting of samples from eastern Slovakia (Laborec, Ulička, and Bodrog Rivers).

The mean genetic *p*‐distance among the sublineages and groups included in our study is 1.81% (range 0.9%–3.1%), while intraspecific and intragroup distances ranged from 0% to 0.6% (Table 8).

**Table 8:** Estimates of evolutionary divergence over sequence pairs between groups

	Group 1	Group 2	Group 3	s‐lin. I	s‐lin. II	s‐lin. IV	s‐lin. V	s‐lin. VI
Group 1	*0.005*	0.003	0.003	0.005	0.003	0.003	0.004	0.003
Group 2	0.013	*0.003*	0.003	0.005	0.003	0.003	0.004	0.003
Group 3	0.013	0.014	*0.003*	0.005	0.003	0.003	0.004	0.003
s‐lin. I	0.029	0.03	0.031	*0.004*	0.004	0.004	0.005	0.004
s‐lin. II	0.017	0.019	0.016	0.027	*0.004*	0.003	0.004	0.003
s‐lin. IV	0.016	0.016	0.017	0.022	0.014	*0.006*	0.004	0.003
s‐lin. V	0.024	0.023	0.024	0.031	0.021	0.018	*0.004*	0.004
s‐lin. VI	0.016	0.017	0.018	0.022	0.014	0.009	0.018	*0.000*

Note

The number of base differences per site from averaging over all sequence pairs between groups is shown. Standard error estimate(s) are shown above the diagonal. Within groups, distances are shown diagonally and written italic.

Abbreviation: s‐lin., sublineage.

### 4.5 Delimitation of golden loaches clades

**Figure 7:**
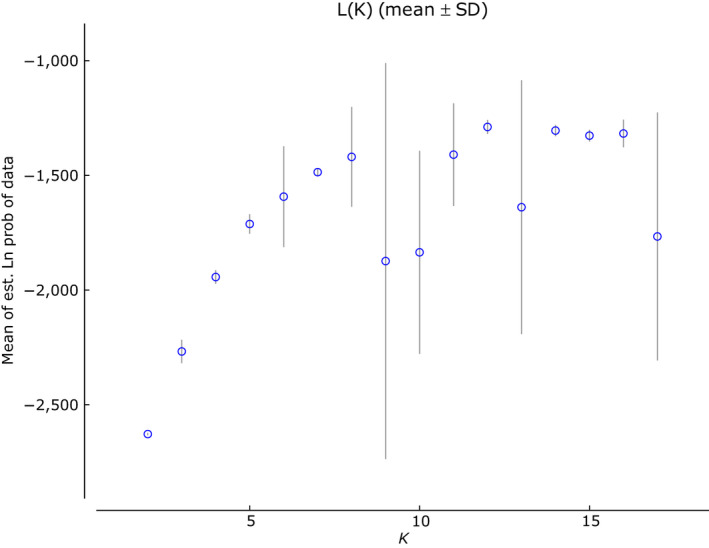
Diagram determining the dependence of the model credibility ("likelihood"; ln Pr(*X*|*K*)) on the growing number of hypothetic groups (*K*)

**Figure 8:**
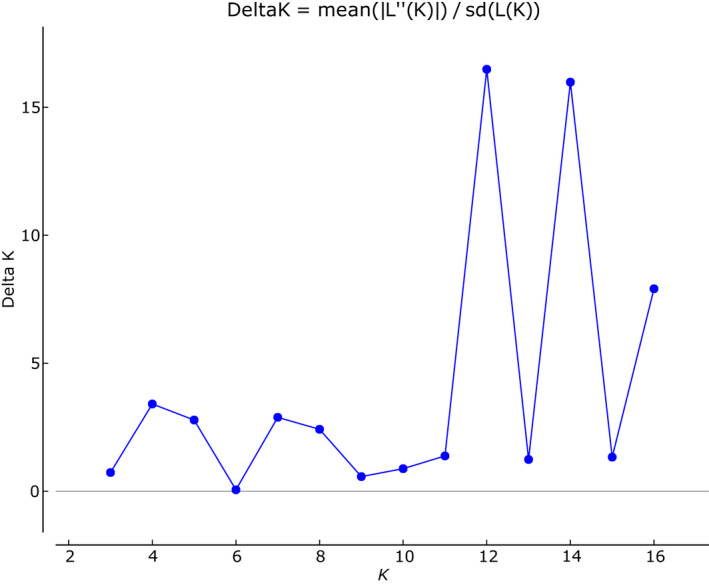
The Δ*K* plot describing the rate of change in the log probability of the data between successive *K* values from 1 to 17. The modal value of this distribution is the true *K*, or the uppermost level of hierarchical structure

**Figure 9:**
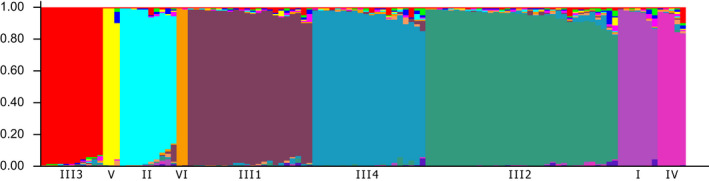
Consensus plot for six independent STRUCTURE analysis runs for *K* = 14. Individual vertical bands depict single individuals within a group, indicating the degree of genotype admixture between sublineages and groups of DB complex

The global cyt *b* dataset was analyzed using the STRUCTURE, bPTP, and mPTP to ascertain the DB complex structure. The uppermost hierarchical level of structure was two clusters at *K* = 12 and *K* = 14 suggested STRUCTURE Harvester analysis (Figures 7 and 8). At both *K*, this analysis indicated nine distinct groups (Figure 9; Figure S1 and Table S4) in agreement with mitochondrial network (Figure 5). The species delimitation methods bPTP and mPTP recognized the same number of candidate species in agreement with sublineages designation (Figures 5 and 6). Both PTP models recognized six candidate species and suggested to modify their names as follows: sublineage I—*S. vallachica* from Romania, sublineage II—*S. balcanica* from North Macedonia and Greece; sublineage III *S. bulgarica* from Danube drainage system; sublineage IV *S. radnensis* from the Mures River system; sublineage V *S. thrakica* from Evros drainage system; and sublineage VI *Sabanejewia* sp. from Mur River in Austria. Both methods also suggested that *S. balcanica* and *S. doiranica* likely correspond to the same species. The level of supports for distinguishing of groups 1–4 within sublineage III was lower or, in case of group 4, none (Table S3). Graphic representation of the mutual relations within DB complex (Figures 5 and 6) is a majority consensus based on the results of the network reconstruction, phylogenetic, and delimitation methods, and therefore, group 4 of sublineage III is not supported more (the hatched network design).

## 5 DISCUSSION

In general, the variation in Slovakian populations observed by ordination analysis (Figure 3) and compared to both samples from type localities for *S. balcanica* (Treska estuary in Skopje, MK) and *S. bulgarica* (Danube River in Vidin, BG) reflects great morphological variation within the genus *Sabanejewia* distribution. Similar large‐scale variation of populations referred to as species *S. balcanica* has been observed in Romania (Bănărescu, 1966; Bănărescu et al., 1972; Iftime, 2002) and Croatia (Buj et al. 2008). Most of the morphometric and meristic and coloration traits exhibit wide range of variability. Our results support the opinion of more or less clinal variation from *bulgarica*‐like (lowland) morphotype to *balcanica*‐like (foothill) one (Iftime, 2002) dispersed mostly in rivers or smaller streams located in submountainous areas or small lower courses of such character (like Blh River). The idea of such variation from lowland to foothill ecomorphs of the same species is also supported by their coloration pattern. It is quite obvious that populations from deeper and larger lower rivers tend to have reduced number of dorsal and lateral spots (Figure 4b). According to Bănărescu et al. (1972), the number of spots decreases in the direction of longitudinal profile of the rivers and depends also on the increasing water depth toward lowland watercourses, what generally matches with our results and results of Fedorčák, Šanda, Stefanov, Mendel, and Koščo (2019). To definitively confirm the hypothesis about clinal variation, it is necessary to carry out the detail research aimed on changes in morphology and coloration within the longitudinal profile of selected rivers in several regions with multiple representatives of this genus. On the other hand, individuals with significant variability in body pigmentation also occur within the same population (Table 6). This fact has been pointed out in several studies (Bajrić, Adrović, Hajdarević, Skenderović, & Tanović, 2018; Balon & Holčík, 1964; Iftime, 2002; Oliva, Balon, & Frank, 1952). According to Oliva et al. (1952), individuals of golden loaches are well matched to the substrate type at a given site by their coloration pattern. Due to the cryptic character of this feature, it can also serve as a form of protection against predators and thus explain the great within groups variability.

Relatively distant position of our populations from Laborec and Vlára Rivers in PCA scatterplot (Figure 3) is mainly due to very short length of barbels of these specimens. These sites were the only ones, where boulders substrate type was dominating. Similarly, short barbels in relation to faster water velocity and stony substrate type were reported by Vasiľeva and Vasiľev (1988, 2019) for population of *Sabanejewia kubanica* in Kura River (Russian Federation). The remaining morphometric characters used in our study have not been shown to be of significant use in distinguishing individual populations. However, the character loadings of PCA (Table 3) revealed several similar identifying features for *bulgaric*a and *balcanica*‐like populations as reported in several previous studies (Bănărescu et al., 1972; Iftime, 2002; Oliva et al., 1952; Sivkov, 1991; Vasiľeva & Vasiľev, 1988). Toward lowland populations, head length (c), preanal (aA), predorsal (aD), and preventral (aV) distance and the length of barbels (lb1, lb2, lb3) increase most significantly. On the other hand, eye diameter (o), caudal peduncle length (lpc), preorbital (prO), and postdorsal (pD) distance are increasing toward foothill morphotype populations. However, in our study we did not confirm the significant difference in body depth (H) reported by several authors (Bănărescu et al., 1972; Iftime, 2002; Kottelat & Freyhof, 2007; Sivkov, 1991) as one the main discriminatory morphometric features. Surprisingly, the highest value of this character was observed in populations from Kysuca and Treska Rivers, that is, typically *balcanica*‐like morphotype (Table 2). In our study, the character of body depth was constantly measured at the origin of dorsal fin. The typical *bulgarica*‐like “hump‐backed” appearance is most pronounced on the body at the level of behind the head. Iftime (2002) however reported that this “hump‐backed” appearance is also considerably variable and is related to breeding conditions. By author, ovigerous females also present distend abdomen, which adds to the overall appearance of body depth. In our case, most of the specimens from Kysuca River were sampled at the beginning of summer, which marks the spawning period for *Sabanejewia* sp. (Juchno & Boroń, 2012), while the other populations were mostly sampled in postspawning period. Therefore, the idea of spawning period impact on the body depth can be explained. Track changes in this and other characters between pre‐ and postspawning period should be a subject of further observations.

In terms of fin rays, our results correspond to previous published data of their numbers within the Central European (Mišík, 1958; Oliva et al., 1952) or Balkan populations (Bajrić et al., 2018; Buj et al. 2008; Sivkov, 1991; Šumer & Povž, 2000). Their number is almost constant in all observed populations, and small deviations between results of individual studies may be due to different counting methods and techniques. The only one more significant difference was observed in a few specimens (Ipeľ, Laborec, Torysa Rivers), in which up to four spines in anal fin were found. So far this number has been reported only by Witkowski (1994) in *S. baltica*. In this case, it is necessary to emphasize the need to use a microscope with a sufficient zoom as well as the need of skin disruption at the location of the fin origin. Some of the spines are of a very short length and also hidden in the skin, making them difficult to observe.

The results of our study prove that the variability within morphology does not reflect groups created from molecular analyses. On the contrary, one of the most important factors affecting the body shape of these small bottom‐dwelling fish is likely represented by local habitat conditions, which are a result of long‐term hydrological conditions at a given site. Therefore, the wide spectrum of morphological variability within the *Sabanejewia* populations in Danube basin could also be understand as a phenotypic heterogeneity among populations caused by diverse environmental characteristics. After analyzing several populations of *Sabanejewia* in Croatia, Buj et al. (2008) came to a conclusion that similar ecological factors are most likely a reason for a morphometrical similarities between populations. The specimens from rivers forming parts of different watersheds but having similar habitat conditions were more uniform than the others.

Our results indicate that morphotype of *S. bulgarica* is bound by its occurrence to larger and deeper lowland rivers with slow velocity and fine substrate bottom. Comparable results have also been reported from the Romania, Bulgaria, or Hungary (Bănărescu et al., 1972; Iftime, 2002; Sivkov, 1991; Stefanov, 2007) as well as from lower courses of the rivers in Central Asia, where specimens of species *S. aurata* have also some lowland morphotype features (reduced body pigmentation and deeper body) similar to *S. bulgarica* description (Bănărescu et al., 1972). The position of specimens from Treska River close to lowland type populations in PCA analysis (Figure 3) may be due to the nature of microhabitats on this site. The sampling locality on this river was situated near the estuary to Vardar River (Marešová et al., 2011), which is relatively large river in this area. Hence, the local ecological conditions on this site can be similar to the lowland streams, where typically *bulgarica*‐like morphotype occurs. Therefore, we consider it appropriate to confirm this theory also through a comprehensive study of *Sabanejewia* populations in the Vardar basin. These conclusions also lead us to claim that body shape of several *Sabanejewia* populations reflects only phenotypic adaptation to diverse habitats. Generally, fish morphology as a manifestation of phenotypic plasticity is a well‐known phenomenon due to diversity of environmental factors (Keeley, Parkinson, & Taylor, 2006; Laporte, Claude, Berrebi, Perret, & Magnan, 2016; Ramler et al. 2016; Senay, Boisclair, & Peres‐Neto, 2014). Phenotypic variability among populations may arise without major genetic differentiation when they occupy heterogeneous habitats across their distribution range (Cheng et al., 2017; Colihueque, Corrales, & Yáñez, 2017).

**Figure 10:**
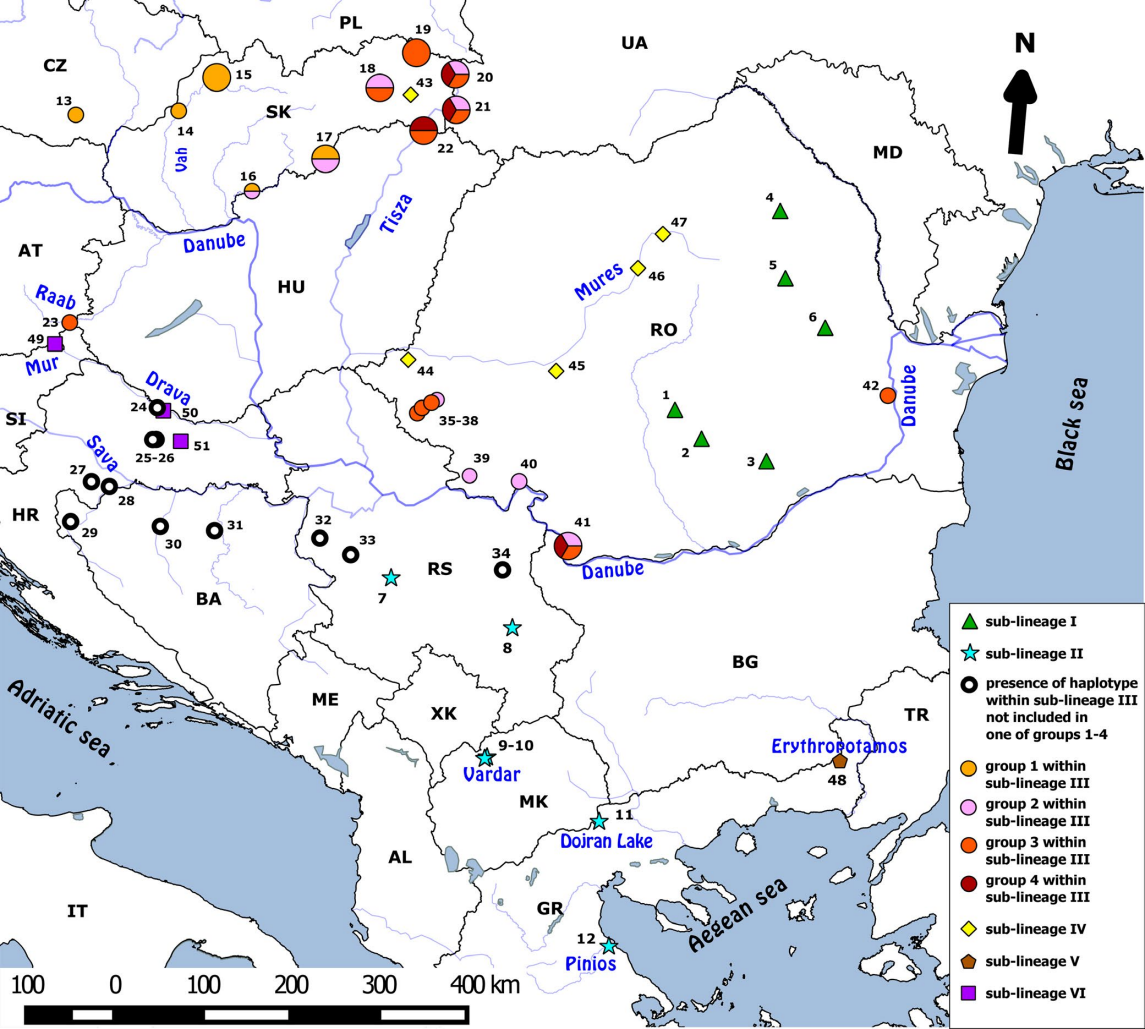
Distribution of *Sabanejewia* sublineages within Danubian–Balkanian complex; larger diagrams represent the original sequences sampled for our study (Abbr.: AL—Albania, AT—Austria, BA—Bosnia and Herzegovina, BG—Bulgaria, CZ—Czech Republic, GR—Greece, HR—Croatia, HU—Hungary, IT—Italy, MD—Republic of Moldova, ME—Montenegro, MK—Republic of North Macedonia, PL—Poland, SI—Slovenia, SK—Slovakia, RO—Romania, RS—Republic of Serbia, XK—Republic of Kosovo, TR—Turkey, UA—Ukraine); data about distribution of DB complex lineages were taken from Buj et al. (2008), Halačka et al. (2017), Marešová et al. (2011) and Perdices et al. (2003);site numbers are listed in Table S1

When comparing two main species concerned of this study (*S. balcanica* vs. *S. bulgarica*) based on molecular analyses, it is necessary to point out the fact that most of previous studies focused on resolving the taxonomic status did not include samples from their *terra typica* (Bartoňová et al., 2008; Perdices et al., 2003; Buj et al. 2008). Our results comprising samples from both of these species have shown that the haplotypes of *S. bulgarica* population from the type locality are spread across almost all haplotype groups in Slovakia and they are also clustered with most of the samples from Danube basin previously considered as species *S. balcanica* (Buj et al. 2008; Halačka, Muška, Mendel, & Vetešník, 2017; Perdices et al., 2003). All phylogenetic and delimitation methods used reliably differentiated the two species and, at the same time, drew our attention to new areas of their occurrence. The new description of distribution of the haplotypes of both the species is in contradiction to the general hypothesis of the dominant position of *S. balcanica* in region of middle Europe and Balkans (Marešová et al., 2011; Perdices et al., 2003). However, populations containing haplotypes of Lineage II (sensu *S. balcanica*) typical for Aegean Sea basin can also be found in the peripheral part of the Danube basin (Marešová et al., 2011) (Figure 10). More precise determination of the border line of occurrence or confirmation of hybrid individuals of both the species will require further investigation especially that performed using the nuclear marker analysis.

The ancient connection between Danube and Vardar River basins in Plio‐Pleistocene period is well documented (Bănărescu, 1992; Economidis & Bănărescu, 1991; Oikonomou, Leprieur, & Leonardos, 2014). Therefore, the occurrence of “Vardar” haplotypes in Danube basin can also be understood as a persistence of ancient polymorphism leading to incomplete isolation of distinct species (Marešová et al., 2011) or recent gene flow between lineages (Bartoňová et al., 2008; Buj et al. 2008). Close phylogenetic relations supporting this claim have also been reported in related genera *Cobitis* (Perdices & Doadrio, 2001) or between barbels species (*Barbus* spp.) (Simonović, Marić, Tošić, Jurlina, & Nikolić, 2018) inhabiting these river basins.

We believe that recent dispersion of variety of mtDNA haplotypes from the type locality of S. bulgarica throughout the Danubian corridor has took place probably during cyclical cold and warm periods in Pleistocene glaciations as reported by Perdices et al. (2003) for the whole DB complex clade. However, much more detailed phylogeographical analysis must be performed for determination of various parameters of distribution, for example, in how many waves, in which numbers of individuals, etc., but this goes beyond the extent of this study. These glaciations played an important role in secondary recolonization from the Danube refuge (Seifertová, Bryja, Vyskočilová, Martínková, & Šimková, 2012; Sommerwerk et al., 2009) leading to low genetic homogenization of freshwater species in this region (Perea et al., 2010). This fact is also most probably the cause of low genetic distances (Table 8) and simultaneous presence of haplotypes of different sublineages of the DB complex at some localities within the Danube basin (Bartoňová et al., 2008; Buj et al. 2008). At present, the relatively short elapsed time from forming the current state of the Danube basin (approximately 700,000 years ago) (Hsü, 1978) and since the establishment of DB complex within *Sabanejewia* genus (Pleistocene period) (Perdices et al., 2003) was not enough to make the genetic distances between lineages more pronounced. However, the blending of haplotypes from type locality of *S. bulgarica* occurring only within the most diverse sublineage III of DB complex is the basis of claim that populations of golden loaches previously referred as species *Sabanejewia balcanica* (Karaman 1922) within Central Europe and Balkan region are closer to naming *Sabanejewia bulgarica* (Drensky, 1928). This is also underlined by fact that morphotype of these fish is very diverse, strongly dependent on local habitat conditions and thus does not allow unambiguous determination based on external morphological features.

## 6 CONCLUSION

Our results demonstrated a high degree of morphological variability among the studied populations of the genus *Sabanejewia*, which is mainly caused by the adaptation of these fish to the ecological conditions on a given habitat. The body shape and coloration pattern in diverse environments reflects local microhabitat conditions and is thus a manifestation of significant phenotypic plasticity. From a phylogenetic point of view, this issue can be characterized as a previously mentioned complex (Perdices et al., 2003) that is currently still in the process of evolution and clear allocation of its species is difficult.

We confirmed that none of the Vardar haplotypes (representing species *S. balcanica*) have been found among Slovakian or other samples included in the sublineage III. Oppositely, haplotypes from Vidin (type locality for *S. bulgarica*) occurred within the sublineage III of Danubian‐Balkanian complex (Perdices et al., 2003) as well as Slovak samples. All these findings form the basis of the claim that populations of golden loaches within the middle part of Danube basin and adjacent regions are closer to name *S. bulgarica*. However, taxonomically there is also Vladykov's description of *Sabanejewia montana* from the mentioned area (Šanda, Vukić, & Švátora, 2010), whose validity could also be reassessed on the basis of further analyses.

In further studies, we suggest a comparison of the biological indicators such as growth differences, fecundity, or more complex molecular studies (nuclear or microsatellite markers) of the DB complex. These could lead to further important knowledge and clarification of this complex issue.

## CONFLICT OF INTEREST

The authors declare no competing interests.

## AUTHOR CONTRIBUTION

**Peter Križek:** Conceptualization (equal); Data curation (lead); Investigation (equal); Methodology (equal); Project administration (lead); Resources (equal); Software (equal); Visualization (equal); Writing‐original draft (equal); Writing‐review & editing (equal). **Jan Mendel:** Data curation (equal); Investigation (equal); Methodology (equal); Software (equal); Writing‐original draft (equal); Writing‐review & editing (equal). **Jakub Fedorčák:** Conceptualization (equal); Data curation (equal); Investigation (equal); Software (supporting); Supervision (supporting); Writing‐review & editing (equal). **Jan Koščo:** Conceptualization (lead); Formal analysis (lead); Funding acquisition (lead); Investigation (supporting); Methodology (supporting); Project administration (equal); Supervision (lead); Writing‐review & editing (equal).

## AUTHOR CONTRIBUTION

P.K., J.F., and J.K. conceived and designed the study, performed sampling, and wrote the paper. P.K. performed laboratory analyses of morphological traits. J.M. performed all molecular analyses. P.K. and J.M. carried out data analyses.

## Supporting information

Fig S1

Table S1

Table S2‐S3

Table S4

## Data Availability

List of all haplotype frequencies GenBank accession numbers, genetic diversity indices: Dryad: https://doi.org/10.5061/dryad.9ghx3fff6
