# Supplementary figures and images for: In the foothill zone—Sabanejewia balcanica (Karaman 1922), in the lowland zone—Sabanejewia bulgarica (Drensky, 1928): Myth or reality?
Source: Ecol Evol. 2020 Jul 3;10(14):7929–47. doi: 10.1002/ece3.6529 (PMC7391564; doi:10.1002/ece3.6529)

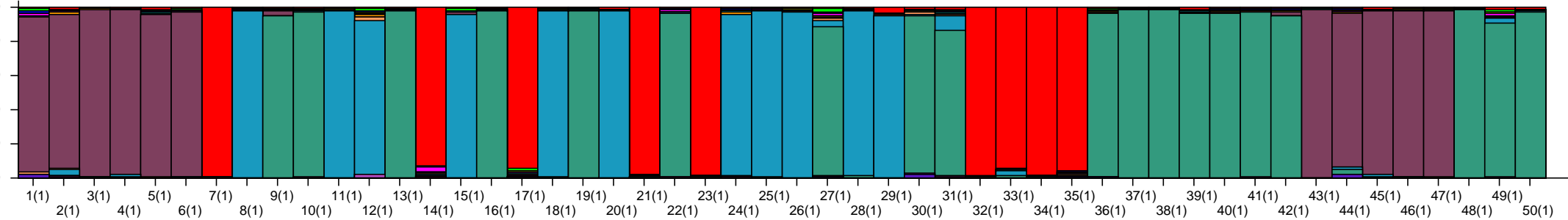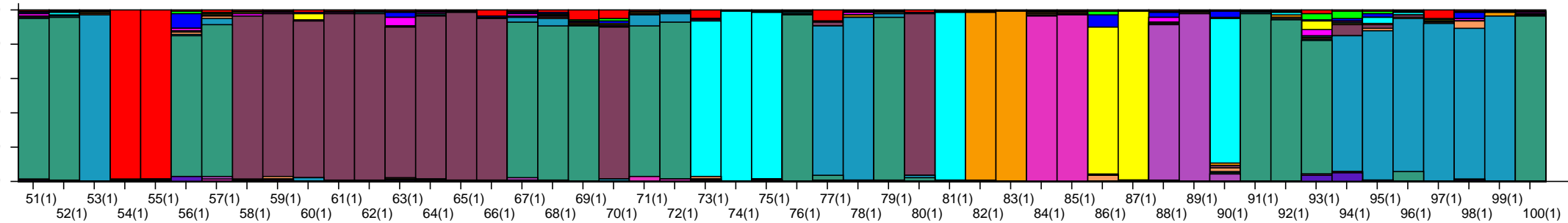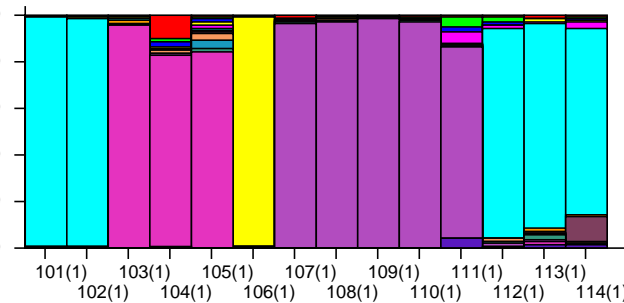

Supplement: Supplementary file 1 — Fig S1 [file ECE3-10-7929-s001.pdf]
