# Supplementary material for: In the foothill zone—Sabanejewia balcanica (Karaman 1922), in the lowland zone—Sabanejewia bulgarica (Drensky, 1928): Myth or reality?
Source: Ecol Evol. 2020 Jul 3;10(14):7929–47. doi: 10.1002/ece3.6529 (PMC7391564; doi:10.1002/ece3.6529)
Supplement: Supplementary file 4 — Table S4 [file ECE3-10-7929-s004.docx]

Tab. S4 STRUCTURE plot for K = 14 - inferred ancestry of individuals (legend of Fig. S1; “group 4“ is highlighted in red; haplotype numbers refer to the numbers in Fig. 5)

Label (%Miss) Pop: Inferred clusters

Sub-lineage: III3 V II VI III1 III4 III2 I IV

1 H1 (0) 1 : 0.007 0.014 0.009 0.001 0.019 0.005 0.001 0.908 0.009 0.001 0.001 0.000 0.000 0.024

2 H2 (0) 1 : 0.010 0.002 0.004 0.003 0.004 0.005 0.009 0.902 0.005 0.037 0.004 0.001 0.013 0.003

3 H5 (0) 1 : 0.002 0.000 0.002 0.001 0.001 0.001 0.001 0.982 0.001 0.001 0.002 0.001 0.001 0.003

4 H3 (0) 1 : 0.002 0.000 0.006 0.001 0.001 0.002 0.002 0.960 0.001 0.014 0.005 0.001 0.003 0.001

5 H4 (0) 1 : 0.009 0.010 0.003 0.001 0.001 0.005 0.007 0.954 0.003 0.002 0.001 0.002 0.001 0.002

6 H6 (0) 1 : 0.006 0.008 0.002 0.002 0.005 0.002 0.001 0.961 0.002 0.004 0.001 0.000 0.003 0.004

7 H7 (0) 1 : 0.986 0.001 0.001 0.000 0.001 0.001 0.001 0.001 0.001 0.001 0.001 0.001 0.003 0.001

8 H8 (0) 1 : 0.001 0.001 0.003 0.002 0.001 0.003 0.003 0.001 0.000 0.979 0.001 0.001 0.001 0.001

9 H9 (0) 1 : 0.006 0.001 0.001 0.004 0.001 0.001 0.001 0.028 0.001 0.001 0.952 0.001 0.001 0.001

10 H9_1 (0) 1 : 0.007 0.001 0.001 0.004 0.001 0.001 0.001 0.001 0.003 0.003 0.969 0.001 0.006 0.001

11 H10 (0) 1 : 0.004 0.001 0.002 0.001 0.003 0.005 0.000 0.002 0.002 0.977 0.001 0.001 0.001 0.001

12 H11 (0) 1 : 0.004 0.013 0.011 0.002 0.000 0.007 0.018 0.002 0.018 0.900 0.001 0.020 0.003 0.002

13 H12 (0) 1 : 0.007 0.000 0.001 0.001 0.001 0.000 0.001 0.001 0.001 0.005 0.982 0.000 0.001 0.000

14 H13 (0) 1 : 0.923 0.006 0.004 0.001 0.025 0.005 0.001 0.001 0.008 0.009 0.006 0.003 0.006 0.003

15 H14 (0) 1 : 0.007 0.009 0.000 0.003 0.001 0.002 0.003 0.011 0.004 0.953 0.000 0.001 0.005 0.001

16 H12_1 (0) 1 : 0.002 0.001 0.003 0.003 0.000 0.001 0.003 0.001 0.003 0.002 0.977 0.001 0.001 0.002

17 H15 (0) 1 : 0.942 0.012 0.002 0.002 0.013 0.003 0.004 0.001 0.010 0.002 0.001 0.002 0.001 0.004

18 H16 (0) 1 : 0.004 0.002 0.001 0.001 0.001 0.001 0.006 0.001 0.001 0.974 0.005 0.001 0.001 0.001

19 H12_2 (0) 1 : 0.005 0.001 0.001 0.000 0.000 0.003 0.002 0.001 0.002 0.001 0.982 0.000 0.001 0.000

20 H17 (0) 1 : 0.009 0.001 0.000 0.000 0.001 0.001 0.001 0.003 0.004 0.974 0.005 0.000 0.001 0.000

21 H18 (0) 1 : 0.977 0.000 0.004 0.000 0.001 0.002 0.001 0.001 0.003 0.002 0.006 0.001 0.000 0.002

22 H19 (0) 1 : 0.004 0.002 0.007 0.000 0.010 0.001 0.001 0.001 0.002 0.007 0.956 0.001 0.002 0.005

23 H18_1 (0) 1 : 0.979 0.001 0.001 0.001 0.006 0.002 0.001 0.001 0.002 0.003 0.002 0.002 0.000 0.000

24 H21 (0) 1 : 0.005 0.001 0.007 0.003 0.005 0.003 0.013 0.000 0.005 0.943 0.001 0.002 0.006 0.006

25 H22 (0) 1 : 0.002 0.002 0.000 0.003 0.001 0.001 0.009 0.002 0.001 0.971 0.002 0.003 0.002 0.001

26 H22_1 (0) 1 : 0.003 0.002 0.002 0.002 0.006 0.002 0.000 0.005 0.001 0.970 0.002 0.003 0.003 0.000

27 H23 (0) 1 : 0.002 0.024 0.004 0.001 0.012 0.005 0.007 0.005 0.012 0.040 0.873 0.002 0.001 0.012

28 H24 (0) 1 : 0.006 0.002 0.001 0.002 0.001 0.001 0.003 0.001 0.001 0.962 0.013 0.001 0.005 0.001

29 H24_1 (0) 1 : 0.030 0.001 0.001 0.001 0.000 0.008 0.002 0.001 0.002 0.949 0.001 0.002 0.002 0.001

30 H25 (0) 1 : 0.011 0.001 0.000 0.000 0.002 0.001 0.007 0.005 0.009 0.013 0.919 0.004 0.004 0.023

31 H26 (0) 1 : 0.011 0.005 0.006 0.004 0.002 0.004 0.007 0.001 0.004 0.086 0.854 0.002 0.008 0.006

32 H27 (0) 1 : 0.985 0.001 0.001 0.000 0.001 0.002 0.001 0.001 0.001 0.001 0.005 0.000 0.001 0.000

33 H28 (0) 1 : 0.939 0.008 0.001 0.001 0.000 0.001 0.000 0.001 0.003 0.031 0.009 0.002 0.002 0.001

34 H27_1 (0) 1 : 0.980 0.001 0.002 0.001 0.001 0.001 0.003 0.003 0.000 0.002 0.002 0.001 0.002 0.000

35 H29 (0) 1 : 0.952 0.002 0.008 0.001 0.004 0.003 0.000 0.007 0.008 0.004 0.001 0.000 0.001 0.006

36 5662 (0) 1 : 0.004 0.005 0.007 0.000 0.004 0.001 0.002 0.001 0.005 0.000 0.960 0.000 0.001 0.010

37 5623 (0) 1 : 0.002 0.001 0.001 0.001 0.001 0.001 0.001 0.003 0.001 0.000 0.984 0.000 0.001 0.003

38 5616 (0) 1 : 0.002 0.001 0.000 0.003 0.000 0.001 0.001 0.001 0.001 0.001 0.986 0.001 0.001 0.001

39 5610 (0) 1 : 0.012 0.005 0.002 0.001 0.001 0.001 0.007 0.001 0.003 0.001 0.963 0.000 0.001 0.001

40 5607 (0) 1 : 0.001 0.005 0.003 0.001 0.009 0.002 0.002 0.005 0.003 0.001 0.965 0.001 0.002 0.002

41 5660 (0) 1 : 0.001 0.005 0.006 0.001 0.003 0.002 0.000 0.002 0.003 0.005 0.964 0.001 0.002 0.005

42 5612 (0) 1 : 0.001 0.001 0.012 0.001 0.005 0.000 0.005 0.018 0.004 0.001 0.945 0.001 0.002 0.002

43 H5_1 (0) 1 : 0.002 0.001 0.001 0.002 0.001 0.004 0.000 0.983 0.001 0.003 0.001 0.000 0.001 0.000

44 5624 (0) 1 : 0.005 0.001 0.007 0.002 0.008 0.004 0.005 0.899 0.004 0.013 0.026 0.002 0.001 0.023

45 5605 (0) 1 : 0.010 0.003 0.000 0.001 0.002 0.001 0.002 0.956 0.000 0.016 0.006 0.001 0.001 0.001

46 5615 (0) 1 : 0.003 0.001 0.003 0.003 0.002 0.003 0.001 0.972 0.001 0.002 0.002 0.003 0.003 0.002

47 5657 (0) 1 : 0.004 0.001 0.002 0.002 0.002 0.001 0.003 0.976 0.002 0.001 0.005 0.000 0.000 0.000

48 H12_3 (0) 1 : 0.003 0.002 0.001 0.001 0.001 0.001 0.001 0.002 0.000 0.000 0.984 0.002 0.001 0.001

49 H41 (0) 1 : 0.013 0.009 0.006 0.007 0.014 0.003 0.000 0.002 0.007 0.029 0.902 0.003 0.002 0.004

50 H12_4 (0) 1 : 0.010 0.001 0.000 0.002 0.001 0.001 0.002 0.001 0.001 0.004 0.973 0.001 0.003 0.001

51 H42 (0) 1 : 0.005 0.007 0.008 0.001 0.009 0.003 0.002 0.001 0.005 0.008 0.936 0.001 0.004 0.009

52 H20 (0) 1 : 0.001 0.001 0.001 0.004 0.001 0.019 0.005 0.002 0.001 0.003 0.954 0.002 0.005 0.002

53 H43 (0) 1 : 0.001 0.002 0.001 0.003 0.000 0.001 0.011 0.002 0.001 0.973 0.001 0.002 0.002 0.001

54 H7_1 (0) 1 : 0.985 0.001 0.001 0.003 0.001 0.001 0.001 0.001 0.001 0.000 0.002 0.000 0.002 0.001

55 H44 (0) 1 : 0.980 0.005 0.001 0.001 0.003 0.001 0.000 0.000 0.002 0.001 0.000 0.001 0.003 0.001

56 H45 (0) 1 : 0.005 0.010 0.087 0.001 0.014 0.000 0.002 0.004 0.018 0.003 0.823 0.000 0.001 0.032

57 H46 (0) 1 : 0.013 0.002 0.003 0.001 0.001 0.001 0.004 0.009 0.015 0.034 0.885 0.017 0.013 0.001

58 6603 (0) 1 : 0.004 0.005 0.003 0.003 0.014 0.001 0.002 0.952 0.004 0.001 0.001 0.004 0.001 0.004

59 6602 (0) 1 : 0.002 0.005 0.005 0.002 0.005 0.002 0.000 0.945 0.015 0.001 0.004 0.002 0.002 0.009

60 5679 (0) 1 : 0.012 0.002 0.004 0.035 0.006 0.001 0.000 0.913 0.000 0.023 0.001 0.001 0.001 0.000

61 5678 (0) 1 : 0.006 0.002 0.004 0.000 0.003 0.000 0.003 0.974 0.001 0.001 0.001 0.001 0.001 0.003

62 5674 (0) 1 : 0.006 0.001 0.001 0.001 0.003 0.006 0.001 0.972 0.003 0.001 0.004 0.000 0.000 0.001

63 5672 (0) 1 : 0.002 0.008 0.031 0.000 0.051 0.000 0.001 0.880 0.011 0.001 0.001 0.001 0.002 0.012

64 5669 (0) 1 : 0.001 0.002 0.006 0.002 0.005 0.007 0.007 0.955 0.000 0.003 0.001 0.002 0.006 0.001

65 6600 (0) 1 : 0.004 0.001 0.001 0.001 0.000 0.001 0.000 0.981 0.001 0.003 0.005 0.001 0.000 0.002

66 H55 (0) 1 : 0.029 0.005 0.002 0.008 0.002 0.001 0.003 0.939 0.002 0.003 0.001 0.001 0.002 0.001

67 H56 (0) 1 : 0.002 0.006 0.008 0.001 0.014 0.003 0.001 0.003 0.004 0.026 0.909 0.012 0.001 0.010

68 H57 (0) 1 : 0.010 0.002 0.005 0.005 0.003 0.001 0.005 0.008 0.006 0.046 0.896 0.001 0.010 0.003

69 H58 (0) 1 : 0.055 0.008 0.002 0.002 0.007 0.004 0.007 0.002 0.002 0.001 0.904 0.001 0.004 0.003

70 H59 (0) 1 : 0.047 0.012 0.019 0.004 0.010 0.000 0.003 0.884 0.003 0.012 0.001 0.002 0.001 0.001

71 H56_1 (0) 1 : 0.005 0.004 0.000 0.001 0.002 0.001 0.003 0.006 0.001 0.063 0.882 0.001 0.026 0.003

72 H56_2 (0) 1 : 0.003 0.003 0.001 0.001 0.001 0.001 0.002 0.004 0.001 0.050 0.913 0.005 0.012 0.003

73 A2793 (0) 1 : 0.044 0.003 0.004 0.002 0.009 0.906 0.002 0.001 0.012 0.002 0.001 0.002 0.011 0.001

74 A2802 (0) 1 : 0.002 0.002 0.001 0.001 0.001 0.987 0.000 0.000 0.003 0.001 0.002 0.001 0.001 0.000

75 A2800 (0) 1 : 0.003 0.004 0.005 0.000 0.002 0.968 0.001 0.001 0.001 0.002 0.001 0.001 0.010 0.001

76  198UB (0) 1 : 0.002 0.001 0.002 0.001 0.002 0.007 0.002 0.005 0.002 0.000 0.972 0.002 0.003 0.001

77 166Danube (0) 1 : 0.062 0.001 0.002 0.001 0.003 0.002 0.000 0.016 0.003 0.873 0.026 0.004 0.004 0.003

78 3 Raab (0) 1 : 0.004 0.001 0.006 0.001 0.010 0.005 0.012 0.001 0.001 0.946 0.000 0.002 0.006 0.004

Label (%Miss) Pop: Inferred clusters

Sub-lineage: III3 V II VI III1 III4 III2 I IV

79 2 Raab (0) 1 : 0.001 0.000 0.001 0.002 0.001 0.001 0.005 0.001 0.008 0.016 0.956 0.000 0.002 0.005

80 Kysuca 1 (0) 1 : 0.013 0.001 0.001 0.001 0.001 0.000 0.002 0.943 0.002 0.009 0.021 0.001 0.005 0.001

81  401G (0) 1 : 0.003 0.004 0.001 0.001 0.000 0.979 0.003 0.001 0.002 0.001 0.001 0.000 0.001 0.003

82 1 MUR (0) 1 : 0.002 0.001 0.000 0.003 0.002 0.003 0.981 0.002 0.000 0.001 0.001 0.002 0.001 0.001

83 3 MUR (0) 1 : 0.001 0.001 0.000 0.002 0.000 0.000 0.991 0.000 0.000 0.001 0.000 0.000 0.001 0.000

84  388CRR (0) 1 : 0.003 0.001 0.001 0.001 0.002 0.002 0.007 0.001 0.009 0.004 0.001 0.001 0.966 0.003

85  368CRR (0) 1 : 0.002 0.001 0.007 0.001 0.002 0.001 0.002 0.002 0.004 0.001 0.002 0.002 0.972 0.002

86 1775G (1) 1 : 0.002 0.025 0.066 0.860 0.008 0.000 0.001 0.001 0.030 0.003 0.000 0.000 0.001 0.001

87 1732CRR (0) 1 : 0.002 0.000 0.002 0.983 0.001 0.001 0.000 0.001 0.001 0.001 0.001 0.000 0.004 0.002

88  127CRU (0) 1 : 0.001 0.013 0.023 0.002 0.031 0.001 0.006 0.001 0.002 0.001 0.001 0.909 0.004 0.005

89 32RU (0) 1 : 0.002 0.002 0.001 0.000 0.001 0.001 0.002 0.000 0.008 0.001 0.001 0.978 0.003 0.001

90  518G (0) 1 : 0.002 0.002 0.037 0.002 0.002 0.847 0.008 0.017 0.024 0.005 0.005 0.045 0.003 0.000

91 519CRU (0) 1 : 0.004 0.001 0.000 0.004 0.001 0.000 0.001 0.001 0.001 0.004 0.981 0.001 0.001 0.000

92  340CRA (0) 1 : 0.006 0.001 0.002 0.001 0.004 0.013 0.012 0.002 0.005 0.005 0.946 0.001 0.002 0.000

93  354CRA (0) 1 : 0.017 0.034 0.007 0.050 0.039 0.005 0.006 0.004 0.007 0.006 0.775 0.004 0.005 0.042

94  358CRA (0) 1 : 0.004 0.041 0.013 0.002 0.011 0.006 0.002 0.065 0.001 0.792 0.002 0.000 0.006 0.053

95  246CRA (0) 1 : 0.002 0.017 0.018 0.001 0.003 0.033 0.005 0.025 0.014 0.868 0.001 0.000 0.001 0.011

96  593CRU (1) 1 : 0.004 0.002 0.001 0.002 0.002 0.015 0.001 0.010 0.006 0.896 0.055 0.001 0.003 0.002

97  261CRA (0) 1 : 0.049 0.006 0.002 0.007 0.003 0.000 0.003 0.001 0.003 0.920 0.001 0.001 0.002 0.001

98  270CRA (0) 1 : 0.001 0.013 0.035 0.001 0.009 0.001 0.001 0.000 0.040 0.881 0.008 0.000 0.001 0.010

99  280CRA (0) 1 : 0.005 0.001 0.000 0.001 0.001 0.003 0.019 0.001 0.001 0.961 0.004 0.001 0.003 0.000

100  218CRB (0) 1 : 0.003 0.001 0.005 0.001 0.006 0.006 0.001 0.001 0.003 0.002 0.969 0.001 0.001 0.000

101  453G (0) 1 : 0.002 0.001 0.001 0.000 0.001 0.982 0.004 0.001 0.001 0.004 0.002 0.001 0.000 0.001

102  467G (0) 1 : 0.003 0.001 0.003 0.001 0.001 0.982 0.000 0.000 0.003 0.001 0.002 0.002 0.001 0.000

103  459CRR (0) 1 : 0.004 0.001 0.000 0.005 0.003 0.002 0.019 0.002 0.001 0.002 0.001 0.001 0.957 0.001

104  369CRR (0) 1 : 0.097 0.016 0.020 0.006 0.007 0.000 0.001 0.003 0.009 0.001 0.002 0.004 0.822 0.009

105  436CRR (0) 1 : 0.004 0.004 0.018 0.010 0.015 0.011 0.009 0.001 0.031 0.038 0.011 0.004 0.843 0.001

106 1776G (0) 1 : 0.002 0.001 0.002 0.983 0.001 0.001 0.001 0.001 0.001 0.003 0.001 0.001 0.000 0.001

107  158RU (0) 1 : 0.009 0.001 0.001 0.001 0.005 0.001 0.001 0.001 0.008 0.000 0.001 0.970 0.000 0.000

108 1RU (0) 1 : 0.002 0.003 0.002 0.001 0.005 0.000 0.000 0.001 0.006 0.003 0.001 0.972 0.000 0.002

109  187CRU (0) 1 : 0.003 0.000 0.001 0.000 0.001 0.001 0.000 0.004 0.001 0.001 0.000 0.985 0.001 0.001

110 81CRU (0) 1 : 0.002 0.006 0.000 0.001 0.004 0.001 0.002 0.000 0.004 0.001 0.001 0.977 0.001 0.001

111  173CRU (0) 1 : 0.002 0.047 0.019 0.001 0.048 0.002 0.003 0.002 0.004 0.001 0.002 0.821 0.001 0.049

112 2803 (0) 1 : 0.002 0.022 0.017 0.001 0.013 0.901 0.001 0.000 0.014 0.000 0.002 0.016 0.001 0.011

113 2805 (0) 1 : 0.011 0.001 0.000 0.011 0.007 0.882 0.013 0.008 0.001 0.006 0.024 0.003 0.014 0.019

114  513G (0) 1 : 0.002 0.006 0.013 0.002 0.028 0.802 0.010 0.108 0.004 0.002 0.003 0.001 0.001 0.017
